# Polycyclic Aromatic Hydrocarbons Bound to PM 2.5 in Urban Coimbatore, India with Emphasis on Source Apportionment

**DOI:** 10.1100/2012/980843

**Published:** 2012-04-29

**Authors:** R. Mohanraj, S. Dhanakumar, G. Solaraj

**Affiliations:** Department of Environmental Management, Bharathidasan University, Tamilnadu Tiruchirappalli 620024, India

## Abstract

Coimbatore is one of the fast growing industrial cities of Southern India with an urban population of 1.9 million. This study attempts to evaluate the trends of airborne fine particulates (PM 2.5) and polyaromatic hydrocarbons (PAH) on them. The PM 2.5 mass was collected in polytetra fluoroethylene filters using fine particulate sampler at monthly intervals during March 2009 to February 2010. PAHs were extracted from PM 2.5 and estimated by high-performance liquid chromatography. It is alarming to note that PM 2.5 values ranged between 27.85 and 165.75 **μ**g/m^3^ and exceeded the air quality standards in many sampling events. The sum of 9 PAHs bound to PM 2.5 in a single sampling event ranged from 4.1 to 1632.3 ng/m^3^. PAH diagnostic ratios and principal component analysis results revealed vehicular emissions and diesel-powered generators as predominant sources of PAH in Coimbatore.

## 1. Introduction

Polycyclic aromatic hydrocarbons (PAHs) among the urban air toxics are of global concern due to their multiple effects on human population. Attention is more importantly on PAHs bound to PM 2.5 and ultrafine fraction of the airborne particulates that are reportedly known for their higher health risk [1, 2]. About 80% of particulate matter in urban environment belongs to the size class of fine (PM 2.5) and ultrafine (PM 0.1) particles [3]. PAHs with two or three benzene rings existed in the vapour phase, whereas PAHs with more than five rings were observed mainly in the particulate phase [4]. PAH size distributions in the atmosphere are influenced by the growth of combustion-generated particles and the variation of PAH adsorption and absorption affinity based on physical characteristics and chemical composition [5, 6]. Higher-molecular-weight (HMW) PAHs, due to their lower vapor pressures, continue to remain glued with fine particles in urban areas where emission sources are high. The PAH “mix” is expected to vary seasonally and geographically as a consequence of changes in emission sources.

Major anthropogenic emission sources of PAHs include biomass burning, coal and petroleum combustion, and coke and metal production [7, 8]. Open burning of biomass, agricultural waste, and municipal solid waste also contribute to atmospheric PAHs. However, vehicular and domestic emissions mainly influence the variation in PAH concentration across the urban to rural gradient [9]. Reportedly higher concentrations of PAHs in urban air have become a major health issue mainly due to their well-known carcinogenic and mutagenic properties [10, 11].

Links between PAH exposure and elevated levels of DNA adducts, mutations, and reproductive defects have strengthened the notorious impact of PAHs [12, 13]. Studies also hint that PAHs can inhibit growth of diatoms and development effects in wildlife. PAHs are also likely to cause transboundary effects and ultimately interfere with the global carbon cycle [14].

In many Asian regions where rapid economic drive is noticed, studies have shown that motor vehicles (especially diesel-engine vehicles), factories, and home heating are the principal sources of atmospheric PAHs and NPAHs [15]. Two wheelers, a popular means of transport in Asian region, are believed to emit particulate PAHs even higher than gasoline- and diesel-powered passenger cars and light- and heavy-duty vehicles [16].

In many Indian cities, rapid urbanization mushrooming of industrialization have triggered the growth of transportation by all means including two wheeler sector. Consequently, these cities suffer from serious air quality problems including rise in PAHs [17, 18]. Annual PAH emissions of India are estimated to be 90 Gg y^−1^ by Zhang and Tao [14]. Reports on fine-particulate-bound PAH are unavailable in many cities. In this context, the present work was aimed to explore the PM 2.5-bound PAH concentration in Coimbatore city and source apportionment. Another objective was to assess the influence of environmental parameters such as temperature, rain, wind speed, and relative humidity on PAH concentration.

## 2. Materials and Methods

### 2.1. Study Area Description

**Figure 1:**
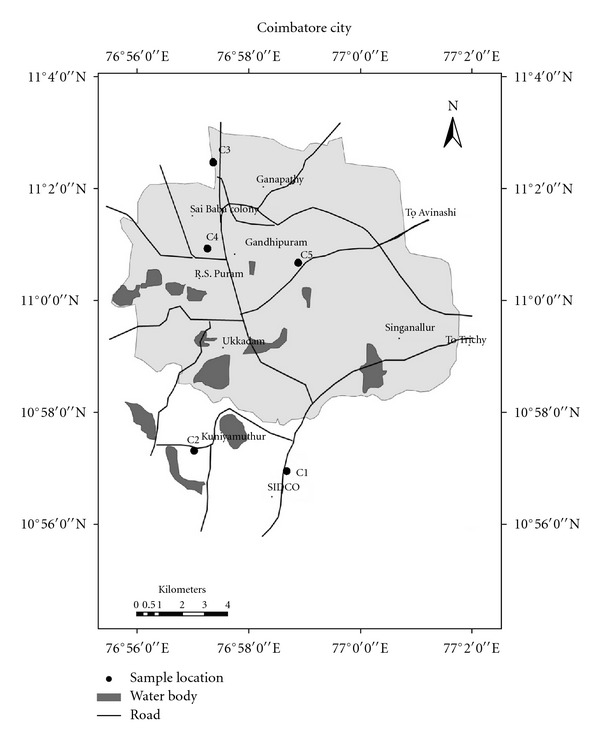
Map showing study sites in Coimbatore.

Coimbatore city and its environs with a population of 1,990,000 occupies the 225th position among the principal urban agglomerations of world as in 2011 (http://www.citypopulation.de/world/Agglomerations.html). Coimbatore city is one of the top ten fastest growing cities of India and one among the top 100 in the world. There are about 25.000 small-scale industries functioning in and around Coimbatore. The number of medium- and large-scale textile mills present in Coimbatore is 312. In the wake of urbanization and industrialization, the number of automobiles is also rising at a similar pace. Among the sources of air pollution, vehicular emissions, industrial emissions, and smoke arising from the garbage dump are prominent in the city. For the current study, five locations namely C1-Small Industries Developmental Corporation (SIDCO), C2-Kuniyamuthur, C3-Kavundampalayam, C4-100 ft road, and C5-Lakshmi mills were selected to represent industrial, suburban residential, mixed commercial and residential, urban commercial, and urban highway, respectively (Figure 1).

The total number of vehicles registered in Coimbatore city as in 2010 had exceeded 1 million. Over the quinquennium ending in 2006, 23 metros posted a compound annual growth rate of 8.3% in the number of total vehicle registrations. Significantly, among the second-tier cities Coimbatore with 12.9% growth rates stood first in Tamilnadu state, India, followed by Madurai city (10.9%) [19]. Coimbatore holds a share of 0.84% and 7.46% of total registered motor vehicles in India and Tamilnadu, respectively. Slower growth in goods vehicle category hints an economic shift from commodity-producing sector (agriculture and industry) towards service sector. High growth in personalized motor vehicles reflects rising per capita income of middle class market competition in automobile sector due globalization coupled with convenient financing options. This proliferation in the personalized mode of transport has evidently increased traffic congestion and particulate air pollution.

### 2.2. Fine Particulate Matter Sample Collection

Fine particulate matter (PM 2.5) sample collection was carried out by Fine Particulate Sampler (Model: APM 550, Manufacturer: Envirotech, New Delhi, India) during March 2009 to February 2010 at five locations once a month in each location for a period of 24 h. The sampling days were different for each location and not simultaneous. Sampler's omnidirectional inlet design prevents the entry of particles greater than 10 microns from the ambient air by a clean aerodynamic cut-point. Further, particles finer than 10 microns in the air stream pass to a second impactor that has an aerodynamic cut-point at 2.5 microns. PM 2.5 particles are finally retained in 47 mm diameter polytetrafluoroethylene (PTFE) filters. The sampler was operated in each station at a constant flow rate of 1 m^3^/hr. After 24 hours of operation, PM 2.5 deposited on the preweighed PTFE filters is estimated gravimetrically and stored in desiccators for PAH investigations.

### 2.3. PAH Analysis

It is assumed that fine particulate matter is uniformly distributed in the PTFE membrane filters. For more precise extraction of PAH from the PTFE filters, several solvents (cyclohexane, benzene, acetonitrile, methanol, dichloromethane) in various combinations were attempted and eventually dichloromethane:methanol (60 : 40) was chosen. A small piece of particulate laden PTFE filter measuring an area of 3.14 cm^2^ was taken for extraction in a dark bottle by adding one mL of dichloromethane:methanol (60 : 40) mixture. Then the filter and solvent mixture was allowed to incubate for half an hour and then ultrasonically extracted for another half an hour [20]. The process was repeated 2-3 times, and final volume was made up to 2 mL. The extracted solution was filtered through 0.2 *μ*m PTFE filter and then redissolved in acetonitrile for HPLC analysis.

HPLC (Manufacturer: Waters, USA) with PAH C18 column (5 *μ*m 4.6 × 250 mm) and fluorescence detector (model: 2475) was used for detection of PAH compounds. Calibrations were performed using 9 individual PAH standards obtained from Supelco, USA, and subsequently samples were analyzed. Chromatographic peaks were identified on the basis of retention time. Fluorescence detector was set at 250 and 425 nm wavelengths, for excitation and emission, respectively. The gradient elution started with 50% water and 50% acetonitrile (up to 5 min), then acetonitrile was increased with a linear gradient to 100% and maintained up to 28 min. The last 12 min elution (28 to 40 min) was performed in 50 : 50 gradients of water and acetonitrile. A total of 9 PAHs were analyzed for this study, namely, phenanthrene (PHENAN), anthracene (ANTH), benzo(a)anthracene (BaA), chrysene (CHRY), benzo(b)fluoranthene (BbF), benzo(k)fluoranthene (BkF), benzo(a)pyrene (BaP), indeno-1, 2, 3-cd pyrene (IcdP), and benzo(ghi)perylene (BghiP). Results of the experiment were subjected to statistics using Statistical Package for Social Scientists (SPSS 11.0). Meteorological data (wind speed, temperature, humidity, and rainfall) obtained from Indian Meteorological Department (IMD) used for further statistical analysis (Table 1). A three-day average inclusive of sampling days and a day prior to it was considered for each meteorological variable.

In order to ensure precision, blank PTFE filters were analyzed for PAH contamination prior to outdoor sample collection. The concentrations of PAH in blank filters were below detection limits for all 9 PAHs investigated. Recovery efficiency was estimated by spike method. Briefly, a half portion of the PTFE filters were spiked with a pre-determined amount of standard PAH solutions. Both the spiked portion of filter and another half of the original filter were subjected to the similar analytical method. In general, recoveries were obtained in the range from 72 to 98% throughout the analysis and values reported were mean of 3 replicates. Reproducibility of the PAH level was calculated based on relative standard deviations (RSDs) by 6-replicate analyses of the same aliquots.

## 3. Results and Discussion

**Figure 2:**
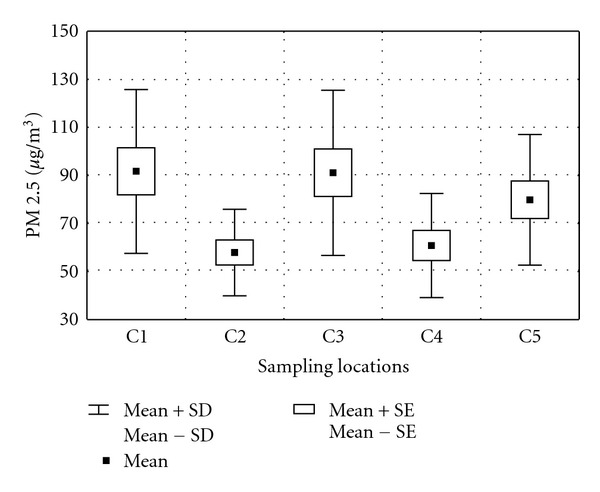
PM 2.5 trends in Coimbatore.

PM 2.5 values in Coimbatore varied between 27.85 and 165.75 *μ*g/m^3^ with an average of 76.28 *μ*g/m^3^ (Figure 2). A maximum of 165.75 *μ*g/m^3^ was recorded in the urban location C3 (Kaundampalayam) during the month of January. Since Kaundampalayam lies at bottleneck edge of the city with roads bound to other towns and touristic locations like Ooty, traffic snarl is a common issue at this exit point. Moreover open garbage dumps present in the vicinity smoldering invariably further exacerbate air pollution levels. Industrial site C1 (SIDCO) with an annual mean of 91.69 *μ*g/m^3^ of PM 2.5 was noted as the most polluted site. It is observed that during the month of June all the stations recorded lower PM values compared to other months indicating a cleansing role of southwest monsoonal rains that are prominent in Coimbatore.

 Lower particulate levels were recorded in suburban residential area C2 (Kuniamuthur), in the range of 36.2 to 88.40 *μ*g/m^3^ with average of 57.93 *μ*g/m^3^. PM 2.5 trend was observed in the following sequence: industrial (SIDCO) > mixed commercial and residential (Kaundampalayam) > urban highway (Lakshmi mills) > urban commercial (100 ft road) > suburban residential (Kuniamuthur). Levels of fine particulate matter in half the number of samples exceeded the 24-hour standard limit of National Ambient Air Quality Standards [34] (NAAQS) (65 *μ*g/m^3^). Analysis of variance of fine particulate matter elucidated statistically significant difference (*P* > 0.05) between sampling location and sampling events. Although no published data is available on PM 2.5 in Coimbatore for comparison, the present observation showed substantially higher concentrations compared to earlier observations of PM 10 indicating the rise in emission sources [35]. 

Descriptive statistics for nine PAH compounds identified in five sampling stations at Coimbatore are shown in Table 2. Annual average of nine PAHs at Kaundampalayam, Lakshmi mills, Kuniamuthur, 100 feet road, and SIDCO was 486.4, 276, 233.2, 175, and 143.48 ng/m^3^, respectively. As mentioned earlier, at Kaundampalayam emissions from traffic and open garbage dump are probable sources for high levels of PAH. In SIDCO industrial area average of 9 PAHs observed during the study period was 143.48 ng/m^3^ without any significant monthly variation compared to other stations. However, BaA value in SIDCO recorded a onetime high value of 292.7 ng/m^3^ during December indicating an unusual source. In SIDCO, engineering and machining industries are mainly involved in production of various accessories and machines required for textile machines, electrical pumps, motors, and other machines, consisting of more than 4000 employees. Plastic industries are involved in manufacturing polythene bags, pipes, joints, and so forth. Main sources of air pollution in SIDCO are probably metal casting operations, cotton textiles, and diesel generators used as power backups. The majority of industries in SIDCO are engaged in metal fabrication and engineering works which obviously did not contribute to elevated levels of PAH although PM 2.5 values are high.

In the urban sampling station at Lakshmi mills, CHRY, BbF, and BghiP were observed as predominant PAHs with annual average of 103.4, 33.2, and 26.6 ng/m^3^, respectively. BghiP has been identified as the tracer of gasoline emissions [36]. Earlier studies had confirmed substantial contribution of BghiP and IcdP from sparking ignition engines [31]. Maximum mean level of BaP, a potent carcinogen, was recorded to be the highest at Lakshmi mills (11.8 ng/m^3^) followed by Kaundampalayam (10.4 ng/m^3^). BaP concentration in Coimbatore city was tenfold higher than the NAAQS standard limit of 1 ng/m^3^. Motorcycles (both 2 strokes and 4 strokes) generally comprise 60–70% of vehicular fleet in Coimbatore city [37]. An earlier study hints that PAH emission factors were the largest for the 2-Stk/Cb motorcycles. Moreover, the 2-Stk/Cb motorcycles had the largest total BaP equivalent emission factor of 10.8 *μ*g/km [38]. Apart from automobile emissions, diesel generator sets used by the industries and medical waste incinerators are also suspected sources of elevated level of BaP [39]. Comparing the total PAH level in Coimbatore with other Indian cities, the present observations were higher than in Chennai and moderately lower than in Kanpur and Kolkata (Table 3).

Domestic cooking fuels also significantly contribute to PAH emissions. A notable percent of the population depends on the following fuels: kerosene, charcoal, wood, straw/shrub/grass, agricultural crop waste, and dung cake for cooking and other domestic purposes [40]. An estimated 61.4 kg/year emission rate of total BaP_eq_ from cooking sources was detected earlier [41]. 

To analyze the extent of seasonal variation in the PAH concentrations, the year was divided into four seasons that is, winter (December to February), summer (March to May), southwest monsoon (June to August), and northeast monsoon (September to November), as per regional meteorological considerations. The mean concentration of total PAHs in five sampling stations to recorded be 481.8, 193.7, 232.3, and 138.9 ng/m^3^ during winter, northeast monsoon, southwest monsoon, and summer season, respectively. Higher PAH levels in winter season suggest the influence of lower temperature and wind speed on stagnation of PAH. On the other hand, the alleviation role of monsoonal rains is also reflected clearly in those sampling months. Summer season recording the lowest levels also confirms photochemical degradation and dispersion of PAHs [42]. Elevated PAH concentration in winter is observed as a common phenomenon in many urban areas [43].

The relationship between PAHs with PM2.5 and meteorological parameters is given in Table 4. Strong positive correlation between IcdP with BbF, BkF, and PHENAN suggests similar source of origin, and negative relationship between these PAHs and temperature infers the role of photolytic degradation [25]. Moderate negative correlation observed between wind speed, rainfall, and individual PAHs suggests dilution and washout effect.

PAH diagnostic ratio computation is one of the most abundantly used methods to identify emission sources based on the concentrations of specific PAH compounds or groups of PAHs. In the current study, site-specific PAH ratios attempted are shown in Table 5. Mean ratio of Ind/(Ind+ BghiP) ranging between 0.26 and 0.66 in all sampling stations indicates likely sources from diesel-driven trucks and catalyst cars. Higher ratio (0.5) of BaA/(CHRY+ BaA) in SIDCO industrial region suggests the use of furnace oil and foundry based furnace operations as source of PAHs. Ratio of BaA/(CHRY+ BaA) observed to be less than 0.3 in other sampling sites in the city suggests catalyst-equipped gasoline cars as a probable source.

Principal component analysis was attempted to identify sources of particulate-bound PAHs in Coimbatore by applying varimax rotation with Kaiser Normalization. The number of significant factors within the data was established by considering only those with an Eigen value >1.0. The degree of association between each variable and each factor was given by its loading on that factor.

**Figure 3:**
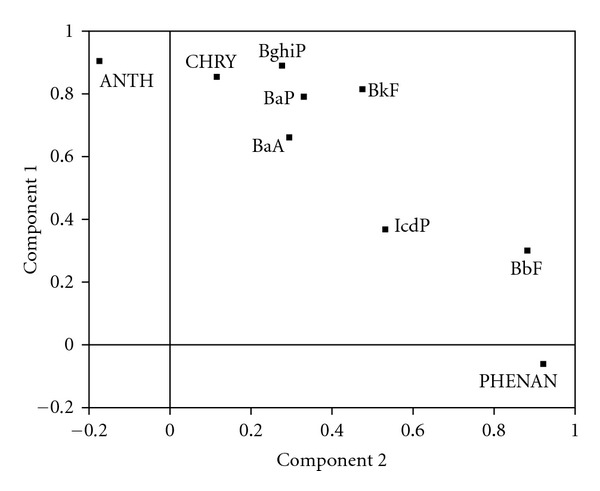
Factor analysis for PAH concentration in Coimbatore city.

In PCA analysis, the total variability of data set is represented by two factors with an account of 74.8% (Figure 3). Factor 1 was loaded with ANTH, CHRY, BkF, BaP, and BghiP, which represents 47.4% of total variance. Most of the factor 1 loadings are tracers of diesel- and gasoline-powered vehicular emissions [5]. Apart from vehicular emissions, diesel generators may also contribute a notable level of these PAHs in Coimbatore city. Therefore, this factor is characteristic of vehicular and diesel generator emissions. Many industrial units in Coimbatore use diesel generators in the event of frequent electricity interruption from public distribution. According to the National Institute of Urban Affairs [44] report, totally 36.579 industrial units are in Coimbatore district, out of which 2.462 units (large industrial units: 138, Medium industrial units 1.082, and small industrial units 1.242) are located in urban limits.

 Factor 2 represents 27.4% of variation with loadings of PHENAN and BbF suggesting combustion of wood materials and backyard burning of biomass as another source [45]. According to the Ministry of Health and Family Welfare [40], nearly 34% of urban and 75% of rural population use wood as a cooking fuel in the state of Tamilnadu. Earlier study also hinted that the ambient PAH concentrations increased significantly during cooking hours and incomplete burning of biomass [46].

## 4. Conclusions

Concentrations of PAHs associated with ambient PM 2.5 were determined at five sites in urban Coimbatore area, for a period of one year. It was found that the level of fine particulate matter (PM 2.5) and its associated polyaromatic hydrocarbons is almost similar to other major metropolitan cities in India. Maximum annual average of total of 9 PAHs was observed to be the highest in urban bottleneck (Kaundampalayam: 486.4 ng/m^3^) than core urban areas (Lakshmi mills: 276 ng/m^3^, 100 feet road: 175 ng/m^3^). The most potent carcinogenic and genotoxic PAH, BaP, was recorded to be the maximum at Lakshmi mills (Annual mean = 11.8 ng/m^3^). The present observation shows that BaP concentration in Coimbatore city was tenfold higher than the NAAQS standard limit of 1 ng/m^3^. Seasonal variation pattern shows significant rise in PAHs concentration during winter compared to other seasons. Source apportionment, analysis reveals vehicular emissions and diesel generator as predominant emission sources of PAH in Coimbatore. Higher ratio (0.5) of BaA/(CHRY+ BaA) in SIDCO industrial region suggests the use of furnace oil and foundry-based furnace operations as source of PAHs. A need for inventory of air trajectory within urban canyon and implementation of good practices such as land use policy and vehicular emission control is imperative.

## Figures and Tables

**Table 1:** Meteorological conditions in Coimbatore during the study period^1^.

Month	Temp. (°C)	Humidity (%)	Wind speed (km/h)	Rainfall (cm)
March	28 ± 1	52 ± 8	7 ± 2	0
April	29 ± 1	57 ± 10	9 ± 2	0
May	28 ± 1	69 ± 6	11 ± 3	3 ± 8
June	27 ± 1	70 ± 7	15 ± 1	0
July	26 ± 1	77 ± 6	16 ± 4	1 ± 2
August	26 ± 1	74 ± 5	14 ± 4	2 ± 7
September	26 ± 1	75 ± 5	13 ± 3	2 ± 7
October	27 ± 1	67 ± 10	8 ± 5	2 ± 6
November	25 ± 1	78 ± 8	6 ± 2	8 ± 17
December	25 ± 1	70 ± 6	7 ± 2	0
January	25 ± 1	59 ± 6	7 ± 2	0
February	27 ± 2	51 ± 6	6 ± 1	0

^1^Source: India Meteorological Department (IMD), New Delhi.

**Table 2:** Descriptive statistics for PAH concentrations in Coimbatore city.

Descriptive statistics	Concentration of PAHs in ng/m^3^									
	PHENAN	ANTH	BaA	CHRY	BbF	BkFi	BaP	BghiP	IcdP	TPAHs
*SIDCO*										
Minimum	BDL	BDL	BDL	BDL	BDL	BDL	0.1	BDL	BDL	4.2
Maximum	17.4	88.3	292.7	113.4	75.5	10.4	18.7	73.3	18.6	619.1
Mean	5.5	25.4	40.1	27.2	15.2	2.1	6.1	17.4	4.5	143.5
*Lakshmi mills*										
Minimum	15.9	BDL	BDL	BDL	3.6	BDL	BDL	4.9	10.9	119.4
Maximum	81.9	40.2	177	195.2	48.4	14.4	21.2	59.1	30	419.2
Mean	38.8	4.5	30.8	103.4	33.2	6.5	11.8	26.6	20.4	276.0
*Kuniamuthur*										
Minimum	BDL	BDL	BDL	0.2	0.2	0.1	BDL	0.1	0.1	4.1
Maximum	157.7	85.0	142.2	312.0	157.7	40.2	20.2	56.5	84.2	744.3
Mean	27.3	12.9	40.3	73.0	30.0	7.7	5.7	16.1	15.2	233.2
*Kavundampalayam*										
Minimum	BDL	BDL	BDL	BDL	BDL	BDL	BDL	BDL	BDL	23.7
Maximum	80.4	278.7	225.0	805.1	43.5	66.3	40.7	98.8	218.8	1632.3
Mean	17.4	43.5	41.9	277.5	16.3	12.5	10.4	30.5	36.4	486.4
*100 feet road*										
Minimum	BDL	BDL	BDL	BDL	BDL	BDL	BDL	6.7	BDL	13.7
Maximum	35.5	55.6	28.5	196.8	18.4	8.0	16.3	37.1	41.5	325.6
Mean	12.1	20.7	8.0	81.4	3.9	3.7	9.1	21.0	15.2	175.0
*Limit of detection (LOD) of PAHs (*μ*g/mL)*										
LOD (*μ*g/mL)	0.0042	0.0014	0.0060	0.0068	0.0056	0.0072	0.0095	0.0088	0.0065	—

*BDL below detectable level.

**Table 3:** Comparison of total PAHs (ng/m^3^) in various cities of the India.

S. No	Total PAHs	Range	City	Reference
1	*∑* 8 PAHs	150–1800	Delhi	[21]
2	*∑* 5 PAHs	244–1481	Chennai	[22]
3	*∑* 5 PAHs	197–2397	Kanpur	[22]
4	*∑* 5 PAHs	284–2114	Kolkata	[22]
5	*∑*12 PAHs	177–1201	Delhi	[23]
5	*∑* 11 PAHs	326–791	Chennai	[24]
6	*∑* 9 PAHs	4.1–1632	Coimbatore	The present study

**Table 4:** Correlation between meteorological parameters and PAH.

	PM 2.5	Temp	Humidity	Wind speed	Rainfall	PHENAN	ANTH	BaA	CHRY	BbF	BkF	BaP	BghiP	IcdP	TPAHs
PM 2.5	1.0														
Temp	−.57	1.0													
Humidity	−.15	.35	1.0												
Wind speed	−.69*	.46	.53	1.0											
Rainfall	.25	.47	.25	−.38	1.0										
PHENAN	−.10	.06	−.24	−.19	−.03	1.0									
ANTH	.39	−.43	−.70*	−.55	−.26	.15	1.0								
BaA	.37	−.60	−.43	−.19	−.57	.17	.71*	1.0							
CHRY	.13	.14	−.44	−.24	.13	.78*	.13	.20	1.0						
BbF	.48	−.57	−.14	−.47	−.16	.68*	.23	.53	.43	1.0					
BkF	.17	−.15	−.37	−.33	−.09	.93**	.09	.27	.83**	.77*	1.0				
BaP	.72*	−.49	−.53	−.72*	.16	.40	.46	.57	.58	.67*	.57	1.0			
BghiP	.63	−.72*	−.17	−.34	−.27	−.03	.14	.20	.07	.43	.27	.30	1.0		
IcdP	.37	−.37	−.26	−.35	−.14	.83**	.13	.43	.75*	.90**	.93**	.67*	.43	1.0	
TPAHs	.20	−.33	−.46	−.29	−.27	.82**	.35	.57	.80**	.77*	.87**	.62	.30	.92**	1.0

*Correlation is significant at the 0.05 level (2-tailed).

**Correlation is significant at the 0.01 level (2-tailed).

**Table 5:** Site-specific diagnostic ratio of PAHs and their probable sources.

Sampling stations	BaP/ BaP+ Chr	BbF/ BkF	BaP/ BghiP	Ind/ BghiP	Ind/ (Ind+ BghiP)	BaA/ (Chr+ BaA)	BghiP/ BaP
SIDCO	0.52	2.98	0.24	0.20	0.26	0.53	2.99
Lakshmi mills	0.09	3.85	0.58	1.15	0.48	0.13	2.11
Kuniyamuthur	0.07	5.49	0.74	1.12	0.44	0.34	2.81
Kaundampalayam	0.05	1.67	1.60	1.99	0.66	0.24	1.16
100 feet road	0.18	0.89	0.53	0.73	0.36	0.12	2.01

Diesel vehicles	>0.73 [25, 26]	>0.5 [27]		~1.0 [28]	0.35–0.70 [29]	0.38–0.64 [30]	1.2–2.2 [31]
Industrial furnaces					0.36–0.57 [32]	0.23–0.89 [32]	0.02–0.06 [32]
Catalyst-equipped cars					0.21–0.22 [30]	0.22–0.55 [33]	2.5–3.3 [31]
